# An Automatic Unmixing Approach to Detect Tissue Chromophores from Multispectral Photoacoustic Imaging

**DOI:** 10.3390/s20113235

**Published:** 2020-06-06

**Authors:** Valeria Grasso, Joost Holthof, Jithin Jose

**Affiliations:** 1 FUJIFILM VisualSonics, 1114 AB Amsterdam, The Netherlands; valeria.grasso@fujifilm.com (V.G.); joost.holthof@fujifilm.com (J.H.); 2 Institute for Animal Science, Hannover Medical School, 30625 Hannover, Germany

**Keywords:** photoacoustic, optoacoustic, spectral imaging, blind source separation, unsupervised unmixing

## Abstract

Multispectral photoacoustic imaging has been widely explored as an emerging tool to visualize and quantify tissue chromophores noninvasively. This modality can capture the spectral absorption signature of prominent tissue chromophores, such as oxygenated, deoxygenated hemoglobin, and other biomarkers in the tissue by using spectral unmixing methods. Currently, most of the reported image processing algorithms use standard unmixing procedures, which include user interaction in the form of providing the expected spectral signatures. For translational research with patients, these types of supervised spectral unmixing can be challenging, as the spectral signature of the tissues can differ with respect to the disease condition. Imaging exogenous contrast agents and accessing their biodistribution can also be problematic, as some of the contrast agents are susceptible to change in spectral properties after the tissue interaction. In this work, we investigated the feasibility of an unsupervised spectral unmixing algorithm to detect and extract the tissue chromophores without any a-priori knowledge and user interaction. The algorithm has been optimized for multispectral photoacoustic imaging in the spectral range of 680–900 nm. The performance of the algorithm has been tested on simulated data, tissue-mimicking phantom, and also on the detection of exogenous contrast agents after the intravenous injection in mice. Our finding shows that the proposed automatic, unsupervised spectral unmixing method has great potential to extract and quantify the tissue chromophores, and this can be used in any wavelength range of the multispectral photoacoustic images.

## 1. Introduction

The accurate detection and quantification of tissue chromophores is vital in molecular imaging, as it can facilitate the early detection, prediction, and monitor the disease conditions. In recent years, multispectral photoacoustic imaging has emerged as a noninvasive tool to visualize the tissue chromophores [1,2,3,4]. The underlying principle of photoacoustic (PA) imaging is based on the conversion of absorbed nanosecond laser pulses into acoustic waves that can be detected just as conventional ultrasound [5,6]. Based on this approach, PA images combine the peculiar optical absorption contrast of the tissue chromophores and the spatial resolution of ultrasound imaging (US). Being a hybrid imaging modality of ultrasound and optical, this multimodal imaging technology can provide anatomical, functional, and molecular information several centimeters deep in the tissues with a resolution up to tens of micrometers. The potential of PA imaging has been demonstrated in various preclinical applications, such as tumor progression and the prediction of tumor recurrence, therapy monitoring, imaging of vasculature, and the biodistribution of the contrast agents [7,8,9,10,11,12,13].

Apart from the preclinical applications, PA imaging is also used in clinical research. In addition to breast cancer monitoring [14,15] and sentinel lymph node imaging [16,17], the PA approach is also used to examine inflammatory bowel disease (IBD) [18] and the temporal arteries in patients with suspected giant cell arteritis (GCA) [19]. To expedite the clinical applications of PA imaging, recently, there has been a lot of focus on developing affordable light sources and the use of this technology in low-resource settings. Xia et al. demonstrated the feasibility of a Light-Emitting Diode (LED) based PA imaging system for the visualization of superficial vasculatures and needle guidance for minimally invasive procedures [20]. Zhu et al. used the LED-based approach to explore more clinical applications, such as diagnosing inflammatory arthritis and assessing peripheral microvascular function in patients [21,22,23].

Although there has been a lot of emphasis on PA hardware development, in the field of affordable settings, data analysis and reconstruction algorithms also play a crucial role in increasing the utility of the technology. Multiwavelength acquisition and spectral image processing is one of the commonly used techniques in PA. Since the optical absorption coefficient of the tissue chromophores varies over the spectrum, multispectral image processing approach can be applied to distinguish and characterize the molecules present in the tissues [24].

In general, the pixel intensity of the multispectral photoacoustic image is proportional to the absorption value of the respective tissue at a specific wavelength. In reality, due to the finite dimension of the pixel (partial volume effect) and the presence of instrumental noise, each spectrum can be a combination of different tissue chromophores. Therefore, it is a challenging task to unmix these signals spectrally and estimate their concentrations. The most common solution to detect the tissue chromophores from multispectral PA imaging is the supervised unmixing [25]. Although this technique yields acceptable results, it requires user interaction to provide the expected source spectral curves as an input to unmix the signals. For translational research with patients, these types of supervised spectral unmixing can be challenging, as the spectral signature of the tissues differs with respect to the disease condition. Imaging exogenous contrast agents and accessing their biodistribution can also be problematic, as some of the contrast agents are susceptible to change in spectral properties after the tissue interaction; thus, the algorithm can forfeit the sensitivity and specificity of imaging.

Hence, an unsupervised unmixing algorithm that can automatically detect the tissue chromophores, without any a-priori knowledge and user interaction, will be optimal, as this can facilitate and improve sensitivity and specificity. Generally, this class of algorithms is referred to as blind source separation (BSS) algorithms, as no a-priori information is required. The study reported by Glatz et al. [26] demonstrated the potential of these approaches to “blindly” extract the oxygenated and deoxygenated hemoglobin absorption spectra from the multispectral photoacoustic images. In the study, they evaluated different unsupervised algorithms, such as multivariate curve resolution analysis (MCR), principal component analysis (PCA), and independent component analysis (ICA) [27,28,29,30,31]. PCA yields an orthogonal transformation that decorrelates the variables. This approach relies upon the hypothesis that the source components are uncorrelated. On the other hand, ICA is based on a different assumption that the source components are maximally independent and non-Gaussian. Recently, Arabul et al. [32] used a similar approach to explore human carotid plaques, in which the ICA blind unmixing approach was constrained non-negatively. Although these approaches demonstrated the potential to detect the tissue chromophores, they suffer from limitations related to the interpretability of the mixed-sign values of their outcomes. Indeed, these aim to fit the training data well but often do not generalize the real and positive data sets.

In this paper, we investigated the possibility of using another blind source separation approach, which is based on non-negative matrix factorization (NNMF) [33]. The concept of NNMF has been widely used in a variety of applications, such as image recognition [34], text classification [35,36], and recommender systems [37]. This approach uses only the non-negative matrices to estimate the prominent components and their spatial distribution, from a linear mixture model. Montcuquet et al. [38] used this approach for in-vivo fluorescent imaging and demonstrated that the positivity condition enhances the convergence and, thus, improves the sensitivity of spectral unmixing [39].

Here, we examine the performance of non-negative matrix factorization (NNMF) to unmix tissue chromophores from multispectral PA images. The algorithm has been optimized to extract the tissue chromophores in the wavelength range of 680–900 nm. We tested the NNMF on synthetic data and on experimental data that mimic the blood vessels. Further, we validated the potential of the approach on an in-vivo study to detect and quantify the endogenous absorbers and exogenous contrast agent accumulation. To our knowledge, this is the first time the NNMF algorithm has been used for PA imaging.

## 2. Non-Negative Matrix Factorization (NNMF)

NNMF is a data decomposition approach, and it is based on the linear mixing model [40]. In this algorithm, the acquired mixed pixel spectra are differentiated into a collection of constituent spectra (called endmembers) and a set of fractional abundance maps. The endmembers represent the pure molecule absorption spectra present in the imaged sample, and these are extracted from the mixed pixel spectra. The maps of abundance at each pixel represent the percentage of each endmember present in that pixel.

Since the acquired spectral images are known, and the rest has to be estimated, the mixed data (multispectral PA images) can be arranged as a matrix $X\in {\mathbb{R}}^{n\times m}$, where ***n*** represents the number of observations (pixels), and ***m*** corresponds to the number of variables per object (different wavelengths). In particular, the unmixing problem can be formulated as a matrix factorization:

(1) X≈WS

where *X* represents the mixed multispectral PA images, *W* the abundance maps, and *S* the source spectra. The dimensions of the matrices *W* and *S* are n×k and k×m, respectively, where ***k*** is the hyperparameter which represents the number of prominent components. 

The NNMF constrained cost function of the optimization problem can be formulated as follows:

(2) $$\left[W,S\right]=\underset{W,S}{\min}\frac{1}{2}∥X-WS{∥}_{F}^{2}$$

(3) $$W_{ij}\geq 0,\;\;S_{ij}\geq 0$$

where, in Equation (2), the defined cost function considers a Frobenius distance between the acquisition *X* and the model *WS* [33]. *W* and *S* are iteratively obtained until both matrices satisfy Equation (1), where the distance defined in the cost function (2) is constrained non-negatively (3). The NNMF learns a parts-based representation of the data, and the whole image is formed as a combination of additive components. The non-negativity constraint is computationally expensive to implement but it can lead to more interpretable data.

To solve the iterative optimization, the multiplicative update rules [41] can be used, and the steps can be defined as:

(4) $$S\;\left(p+1\right)=S\left(p\right)⊗\frac{\left(W^{T}\cdot X\right)}{\left(W^{T}W\cdot S\right)}$$

(5) $$W\left(p+1\right)=W\left(p\right)⊗\frac{\left(X\cdot S^{T}\right)}{\left(W\cdot S\cdot S^{T}\right)}$$

where ***p*** is the iteration step, and the operations of ⊗ and division in (4) and (5) are considered element by element. Each component is estimated only up to a multiplying scale factor. Hence, the factorization problem does not have a unique solution, where *WS* is a lower-rank approximation of *X.*

## 3. Experimental Methods

### 3.1. Simulated Multispectral PA Images 

To evaluate the performance of the NNMF algorithm, simulation studies were performed by using a synthetic data set. The synthetic data set contains implemented photoacoustic spectral images within the wavelength range of 680–900 nm, with a step size of 5 nm. Each image at the respective wavelength contains 400 × 600 pixels. From the photoacoustic signal generation, it is evident that the PA signal is not only proportional to the absorption coefficient but also depends on the local fluence. The light fluence generally decreases with depth, and thus degrades the image uniformity, causing spatial fluence variations within the tissue. Consequently, the fluence compensation is significant for quantitative spectral imaging. Since the main focus of the simulation was to test the unmixing algorithm and its accuracy to detect the spectral signature of the prominent components, in this study, the local fluence was assumed to be constant.

**Figure 1:**
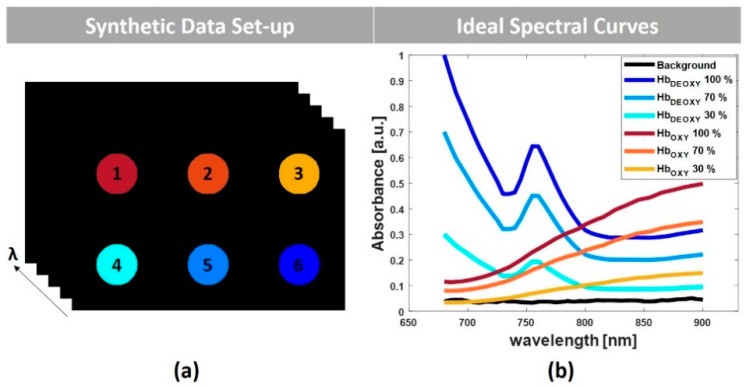
(**a**) Schematic representation of the simulated multispectral photoacoustic (PA) images with six inclusions, (**b**) ideal spectral curves of the inclusions.

Figure 1a depicts the schematic of the 2-dimensional (2-D) data set with six homogeneous inclusions. The inclusions mimic the cross section of blood vessels with different concentrations of oxyhemoglobin and deoxyhemoglobin. The inclusions 1, 2, and 3 contain oxyhemoglobin at 100%, 70%, and 30% of the intensity, respectively. Conversely, the inclusions 4, 5, and 6 include deoxyhemoglobin at 30%, 70%, and 100% of the intensity. Figure 1b shows the theoretical absorption spectra [42] expected from these inclusions, in addition to the background tissue absorption. To mimic the experimental conditions, a positive Gaussian distribution of noise (mean=0.04; std=0.1% ; SNR=30 dB) was also added to the respective data set. 

### 3.2. Experimental Set-Up and Tissue-Mimicking Vessel Phantom 

**Figure 2:**
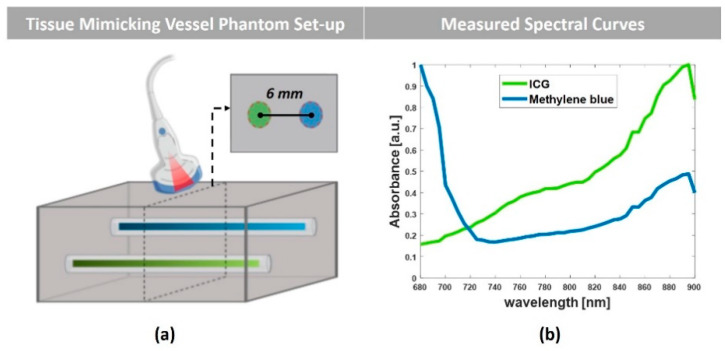
(**a**) Schematic of the tissue-mimicking vessel phantom. (**b**) The PA absorbance spectral graph of the agents measured by using VevoLab.

In addition to the simulation studies, experiments were performed by using Vevo LAZR-X photoacoustic image technology (FUJIFILM VisualSonics, Inc., Toronto, ON, Canada), as described elsewhere [43]. Vevo Phantom (FUJIFILM VisualSonics, Inc., Toronto, ON, Canada) containing two capillary tubes filled with Indocyanine Green (ICG, PULSION Verwaltungs, GmbH) and Methylene Blue (MB, Sigma-Aldrich), was used to mimic the blood vessels in the tissue. Transparent polyethylene (PE) tubes (SAI Infusion Technologies, Lake Villa, IL, USA), with an inner diameter of 15 µm and an outer diameter of 33 µm, were used. The tubes were positioned at a reciprocal distance of 6 mm and fixed at the same depth of 14 mm from the surface of the transducer. Demineralized water was used as a coupling medium, and the multispectral PA images were obtained in the wavelength range of 680–900 nm, with a step size of 5 nm. Figure 2a shows the schematic of the phantom and the experimental set-up. A 256-element linear array transducer with a central frequency of 21 MHz (MX250), including the integrated light delivery fibers from the sides of the transducer, was used to acquire the PA images. The transducer was aligned perpendicular to the capillary tubes, and the cross-sectional image of the tubes was acquired throughout the wavelength range. 3-Dimensional (3-D) data sets were also collected by linearly translating the transducer with a stepper motor over the capillary tubes while capturing cross-sectional 2-D slices. Figure 2b shows the photoacoustic spectra measured from the capillary tubes by using the Spectro-Mode in the VevoLab software (FUJIFILM VisualSonics, Inc., Toronto, ON, Canada). In the measurement tool, the system allows the user to select the region of interest (ROI) and calculate the average intensity of the photoacoustic signal at different wavelengths. Although this is not quantitative and the values are in arbitrary units, it can provide the spectral absorption trend of the agents in the respective wavelength range. 

### 3.3. In-Vivo Study 

Further, in-vivo animal experiments were performed to evaluate the feasibility of the NNMF data analysis on multispectral PA Imaging. The animal experiments were performed at the FUJIFILM Sonosite/VisualSonics facility in Amsterdam. The animal protocols used in this work were evaluated and approved by the Animal Use and Ethics Committee (CEUA) of The Netherlands (Protocol AVD2450020173644). They are in accordance with FELASA guidelines and the National Law for Laboratory Animal Experimentation (Law No. 18.611). The experiments were performed by using the same apparatus (Vevo LAZR-X) used for the phantom studies. A CD-1 female mouse model (Envigo, Horst, the Netherlands) was used for the experiments. The animal was anesthetized with isoflurane and placed on the animal imaging platform of the Vevo LAZR-X system, where temperature, heart rate, and respiration rate were monitored in real time. During the experiments, anesthesia was maintained using a vaporized isoflurane (1 L/min of oxygen and 0.75% isoflurane) gas system. The animal was positioned in right lateral recumbency, and the transducer was aligned perpendicularly. The kidney–spleen area of the animal was imaged before and after the intravenous injection of ICG. To obtain a concentration of 800 μM, 25-mg vial of ICG (PULSION Verwaltungs, GmbH) was resuspended in sterile water. With the help of an infusion pump (flowrate of 15 μL/sec), 80 μL of ICG was injected into the tail vein, and multispectral PA images were acquired in the wavelength range of 680–900 nm.

## 4. Results and Discussion

### 4.1. Simulated Multispectral PA Images 

**Figure 3:**
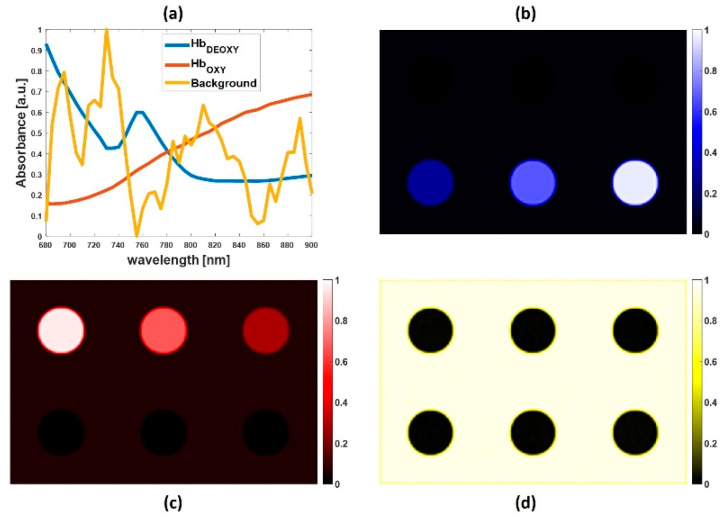
(**a**) Source spectra of oxyhemoglobin, deoxyhemoglobin, and background extracted by the non-negative matrix factorization (NNMF) algorithm. Abundance maps of (**b**) deoxyhemoglobin, (**c**) oxyhemoglobin, and (**d**) background.

Figure 3 shows the main component spectra (a) and the respective abundance maps (b, c, and d) extracted from the synthetic PA data set by using the NNMF algorithm. The obtained spectral graphs (Figure 3a) show that the prominent absorbers present within the inclusions consist of two different endmembers: oxyhemoglobin and deoxyhemoglobin. As expected from the synthetic data, the oxyhemoglobin is mainly distributed on the first row of inclusions, with decreasing intensity from left to right (Figure 3c). The deoxyhemoglobin is present in the second row of inclusions, with increasing intensity from left to right (Figure 3b). Figure 3d displays the spatial distribution of the detected third component (named as background in Figure 3a), which is principally present in the region around the inclusions.

NNMF appears to be an accurate method, yielding an explicit unmixing of specific tissue biomarkers, such as oxyhemoglobin and deoxyhemoglobin, and extracting the respective spectral signatures. The calculated Pearson correlation coefficient between the ideal spectra of the oxy and deoxyhemoglobin, and the extracted spectra was equal to 1 (Table 1). This confirms that NNMF can provide encouraging results on extracting tissue chromophores from multispectral PA images.

**Figure 4:**
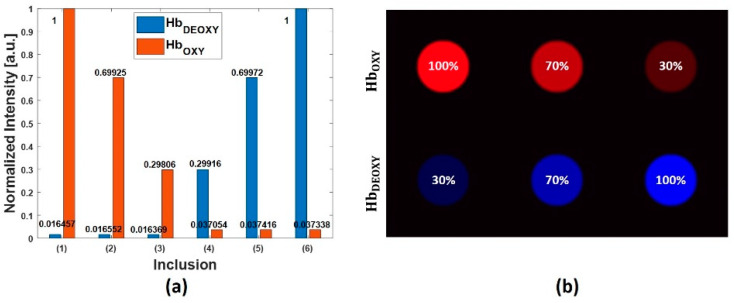
(**a**) Quantitative evaluation of the prominent source components (oxyhemoglobin and deoxyhemoglobin), per each circular region of the synthetic phantom. (**b**) Overlapped abundance maps of oxyhemoglobin and deoxyhemoglobin.

Further, we investigated the performance of the NNMF approach to quantify the extracted prominent components. Figure 4a shows a graph with the quantitation of the source components (oxyhemoglobin, deoxyhemoglobin) per each circular inclusion of the synthetic data. Considering the synthetic data: inclusions 1, 2, and 3 present approximatively a decreasing content of oxyhemoglobin, and the regions 4, 5, and 6 present an increasing content of deoxyhemoglobin. In the graph, the variations are in the range of zero to one, and it is evident that these normalized intensities are matching the expected proportion of the components. Figure 4b shows the overlapped abundance maps of oxyhemoglobin and deoxyhemoglobin, and it also confirms the expected distribution of the prominent components.

### 4.2. Tissue-Mimicking Vessel Phantom 

**Figure 5:**
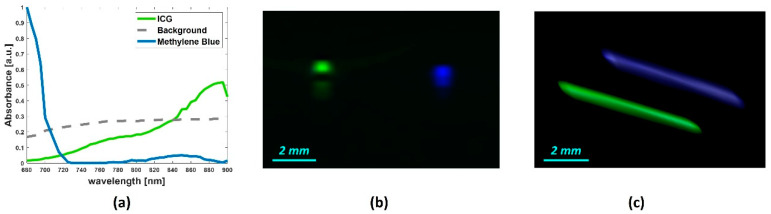
(**a**) Spectral absorption curves of the detected source components by NNMF, from 2-D spectral PA images of the tissue-mimicking vessel phantom. The overlapped abundance 2-D maps (**b**) and 3-D maps (**c**) of the detected source components: ICG and MB.

Figure 5 shows the prominent spectral curves (a) and the abundance maps of the dyes in the capillary tubes, obtained by using the NNMF algorithm. The abundance maps overlapped in Figure 5b correspond to the 2-D spatial distribution of the contrast agents (ICG in green and MB in blue). Figure 5c shows the unmixing of the capillary tubes in 3-D, where the total imaged range was 6.5 mm with a step size of 200 µm. The Pearson correlation coefficient was evaluated between the extracted spectral curves, by using the NNMF, and the spectra measured by using the Spectro-Mode in the VevoLab. 

Table 1 reports the correlation coefficients measured for the ICG and methylene blue. The correlation value obtained for the methylene blue was 0.8344, and it was comparatively lower to the value obtained for the ICG, which was 0.9943. This could be due to the experimental conditions, as the tubes are located adjacently, into the phantom chamber. Hence, due to the short distance, the ICG that has an absorption peak at around 880 nm could influence the absorption spectrum of the MB. The graph reported in Figure 2b supports this assumption, as the MB spectrum shows an additional peak at around 880 nm. This may entail that measured spectral curves in Figure 2b are not the pure agent spectra. On the other hand, the spectra extracted by using NNMF are in accordance with the expected spectral signatures, and it shows promising unmixing performance. 

### 4.3. In-Vivo Study

**Figure 6:**
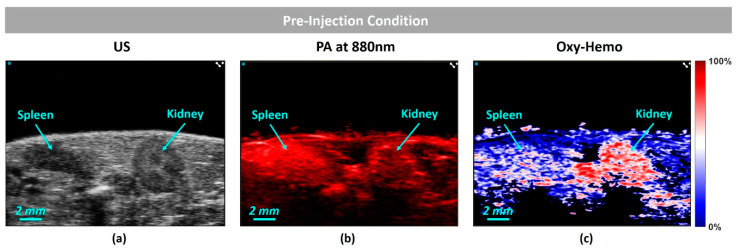
Pre-injection conditions: (**a**) ultrasound (US) image of the kidney–spleen view; respectively (**b**) photoacoustic (PA) image obtained at 880nm, and (**c**) $SO_{2}$ map.

Figure 6a shows a high-resolution ultrasound (US) image of the kidney–spleen region and (b) is the PA image obtained at 880 nm. Figure 6c shows the oxygen saturation ($SO_{2}$) map, obtained before the contrast agent injection. To obtain the oxygen saturation map, we have followed the algorithm reported by Needles et al. [5], where the pixel values of the image are in the range of 0% (lower oxygenation) to 100% (higher oxygenation).

**Figure 7:**
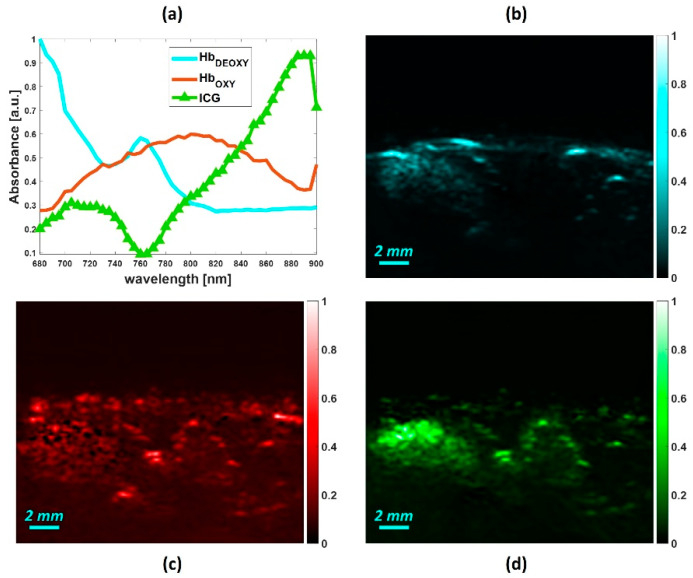
Post-injection conditions: (**a**) spectral signature of the endmembers obtained by using NNMF and abundance distribution maps of (**b**) deoxyhemoglobin, (**c**) oxyhemoglobin, and (**d**) ICG.

Figure 7 shows the post-ICG injection condition. Figure 7a displays the source absorption spectra extracted by the NNMF approach. As expected, in the post-injection condition, the NNMF extracted the typical absorption spectral curve of the ICG, in addition to the endogenous chromophores such as oxy and deoxyhemoglobin spectra. The extracted spectrum of the ICG appears slightly different than the vessel mimicking phantom. Although the peak absorption was at 880 nm, the ICG spectrum at the lower wavelengths was altered. This could be due to the ICG interaction with other chromophores within the tissues. Figure 7 also shows the abundance maps of deoxyhemoglobin (b), oxyhemoglobin (c), and ICG (d). In the ICG map, it is evident that the dye is mostly accumulated in the spleen region. This is in accordance with the biodistribution of the ICG, as the kinetic of the kidney is much faster than the spleen [44].

## 5. Conclusions

In summary, we investigated the spectral decomposition of various tissue biomarkers from multispectral PA images. In particular, we have explored the performance of an unsupervised spectral unmixing algorithm, NNMF, in the wavelength range of 680–900 nm. Considering the evidence obtained from the initial results, the NNMF can extract both endogenous and exogenous agents from the multispectral PA data. The unmixing results obtained from the simulation studies performed on synthetic data revealed a high correlation with the expected spectra, and also yielded the quantification of the chromophores. The experiment performed on the tissue-mimicking phantom also supported the results obtained on the synthetic data. Indeed, the NNMF showed promising unmixing performance, allowing the accurate detection of ICG and MB, by eliminating the spectral influence of the other dyes. The in-vivo experiments in the animal model showed the detection of a contrast agent signature, accounting for the spectral variations that may ensue due to tissue–dye interactions. The NNMF can also provide the maps of abundance distribution of contrast agents in different anatomical targets, facilitating in-vivo biodistribution and kinetics studies. Since the algorithm facilitates the automatic unmixing of the tissue chromophores, without any a-priori knowledge about the source components and user interactions, it is easy to adapt, and promising for data-driven studies in multispectral photoacoustic imaging.

In the current algorithm, we have used the time gain compensation (TGC) approach to overcome the PA signal attenuation through depth. Although the approach is not quantitative, it gives improved results. Recent studies demonstrate that taking into account the fluence variance [45,46] can overcome the quantification limits of photoacoustic imaging. Therefore, in future studies, we will investigate the corruption effects of the fluence variation, the finite size, and band-limited frequency response of the detectors, to consider the respective changes on the absorption spectra. Besides, we will expand the current wavelength range of 680–900 nm to the far infrared (FIR), as this may entail the detection of less prominent tissue chromophores, such as melanin, lipids, and collagens.

In conclusion, to the best of our knowledge, this would be the first time that NNMF was used for unmixing multispectral PA imaging. The obtained results confirmed that the NNMF algorithm automatically and accurately detects the component spectra. This proves that the imposed positivity constraints, to the source spectra and abundance distribution maps, are appropriate requirements to unmix tissue chromophores from multispectral PA images.

## Figures and Tables

**Table 1:** Correlation values between the extracted source components by using the NNMF unmixing approach, and the respective absorption spectral curves used as a reference.

Data Set-Up	Chromophores	Correlation Value
Synthetic data	Oxyhemoglobin	1
	Deoxyhemoglobin	1
Vessel mimicking phantom	ICG	0.9943
	Methylene Blue	0.8344
